# Orally administered docetaxel-loaded chitosan-decorated cationic PLGA nanoparticles for intestinal tumors: formulation, comprehensive in vitro characterization, and release kinetics

**DOI:** 10.3762/bjnano.13.115

**Published:** 2022-11-23

**Authors:** Sedat Ünal, Osman Doğan, Yeşim Aktaş

**Affiliations:** 1 Department of Pharmaceutical Technology, Erciyes University Faculty of Pharmacy, Kayseri, Turkey; 2 Department of Bioengineering, Faculty of Life and Natural Science, Abdullah Gül University, Kayseri, Turkey

**Keywords:** chitosan, docetaxel, intestinal tumors, oral drug delivery, PLGA

## Abstract

Intestinal cancers are the third most lethal cancers globally, beginning as polyps in the intestine and spreading with a severe metastatic tendency. Chemotherapeutic drugs used in the treatment of intestinal tumors are usually formulated for parenteral administration due to poor solubility and bioavailability problems. Pharmaceutically, clinical failure due to a drug’s wide biodistribution and non-selective toxicity is one of the major challenges of chemotherapy. In addition, parenteral drug administration in chronic diseases that require long-term drug use, such as intestinal tumors, is challenging in terms of patient compliance and poses a burden in terms of health economy. Especially in the field of chemotherapy research, oral chemotherapy is a subject that has been intensively researched in recent years, and developments in this field will provide serious breakthroughs both scientifically and socially. Development of orally applicable nanodrug formulations that can act against diseases seen in the distant region of the gastrointestinal tract (GIT), such as intestinal tumor, brings with it a series of difficulties depending on the drug and/or GIT physiology. The aim of this study is to develop an oral nanoparticle drug delivery system loaded with docetaxel (DCX) as an anticancer drug, using poly(lactic-*co*-glycolic acid) (PLGA) as nanoparticle material, and modified with chitosan (CS) to gain mucoadhesive properties. In this context, an innovative nanoparticle formulation that can protect orally administered DCX from GIT conditions and deliver the drug to the intestinal tumoral region by accumulating in mucus has been designed. For this purpose, DCX-PLGA nanoparticles (NPs) and CS/DCX-PLGA NPs were prepared, and their in vitro characteristics were elucidated. Nanoparticles around 250–300 nm were obtained. DCX-PLGA NPs had positive surface charge with CS coating. The formulations have the potential to deliver the encapsulated drug to the bowel according to the in vitro release studies in three different simulated GIT fluids for approximately 72 h. Mucin interaction and penetration into the artificial mucus layer were also investigated in detail, and the mucoadhesive and mucus-penetration characteristics of the formulations were examined. Furthermore, in vitro release kinetic studies of the NPs were elucidated. DCX-PLGA NPs were found to be compatible with the Weibull model, and CS/DCX-PLGA NPs were found to be compatible with the Peppas–Sahlin model. Within the scope of in vitro cytotoxicity studies, the drug-loaded NPs showed significantly higher cytotoxicity than a DCX solution on the HT-29 colon cell line, and CS/DCX-PLGA showed the highest cytotoxicity (*p* < 0.05). According to the permeability studies on the Caco-2 cell line, the CS/DCX-PLGA formulation increased permeability by 383% compared to free DCX (*p* < 0.05). In the light of all results, CS/DCX-PLGA NPs can offer a promising and innovative approach as an oral anticancer drug-loaded nanoformulation for intestinal tumors.

## Introduction

Cancer is one of the most common chronic diseases in the world, characterized by the uncontrolled proliferation and spread of cells [1]. To date, effective and safe treatment approaches for cancer treatment have not been fully developed, and researchers are still working on this issue. For many types of cancer, selective, targeted, and definitive treatment methods have not been developed yet. Colon carcinomas are the fourth most frequently diagnosed cancer type and still the third most leading cause of cancer-related death. They are among the most serious types of cancer affecting humanity every year on a global scale [2–3]. Colon carcinoma, which starts as polyps on the inner surface of the colon, is a malignancy that envelops the colon mucosa over time, progresses by invading the colon tissues, and is characterized by severe metastasis tendency. Symptoms such as bowel bleeds, constipation, and severe abdominal pain are common in cases of colon cancer. In the advanced stage, the tendency to metastasize to vital organs such as the liver is a serious complication and adversely affects the clinical course of the patient and shortens the average life span [4–5]. The two most significant factors influencing survival in patients with colorectal cancer are tumor recurrence and propensities for distant metastasis. As cancer is connected with significant morbidity and mortality, investigations are still being conducted to find new diagnostic and therapeutic approaches. The absence of an efficient oral chemotherapy is one of the biggest obstacles in cancer treatment worldwide [6].

Various formulation approaches have been used for many years to provide higher drug concentration in colon and less systemic side effects [7–9]. However, each brings its own advantages and disadvantages, and an effective formulation for colon carcinomas has not been developed yet. From this point of view, novel drug delivery systems and nanoparticular drug delivery systems are considered and evaluated as trends and promising approaches in the treatment of colon carcinomas as well as of many other diseases [10–13].

Cancer chemotherapy is still mostly administered parenterally, which is a negative factor in terms of patient comfort. Also, non-specific wide biodistribution of the drug after parenteral administration can cause adverse effects in healthy cells, serious side effects, and decrease in clinical efficacy [14–15]. Because of its simplicity, oral drug administration is the most popular method, particularly for chronic patients who require long-term treatment. Since it is painless and self-administered, there is no need for a medical facility or health professional for each dose. It is less stressful and more affordable for the patient [16]. Considering that cancer is a chronic disease and that the person needs long-term treatment, oral formulations for cancer chemotherapy are still an issue of interest. However, the occurrence of colon carcinomas in the most distant region of the gastrointestinal tract makes the development of oral formulations for colon carcinomas more difficult than that of other formulations [10]. Considering multiple factors such as the variable pH of the gastrointestinal tract, enzymatic destructive environment, and transit time through the gastrointestinal tract, the ability of an orally administered formulation to reach colon carcinomas stably and effectively necessitates a serious pharmaceutical formulation study and a comprehensive evaluation. It is possible to overcome the multiple GIT-related barriers through oral administration of nanoparticulate drug delivery systems. From this point of view, polymeric nanoparticles (NPs) are promising in the development of an oral formulation for colon carcinomas. While it protects the drug from various destructive effects of GIT with its polymeric protective structure, with the help of some modifications such as surface modifications, it allows the nanoformulation to exhibit locally higher concentrations in the colon [13–14,16–18]. Physiologically specific factors in the tumor microenvironment, such as increased negatively charged mucin, decreased pH value, and increased temperature, may provide design clues for mucoadhesive polymeric nanoparticles that have a potential to exhibit higher drug release or help to alleviate colorectal tumor in colon region [11,19–20].

PLGA is a physiologically biocompatible and biodegradable polymer approved by the FDA, which can be synthesized as a copolymer of lactic and glycolic acids at various monomer ratios [21]. With its chemical structure suitable for the preparation of nanoparticulate drug delivery systems and its polymeric structure suitable for drug release profile designs, it is frequently used in research especially in the development of nanoparticulate drug formulations [17,22–24]. Chitosan (CS) is a common biocompatible polymer used extensively in drug delivery applications as a vehicle for drugs, proteins, and nucleic acids. Also, it is used as a coating polymer in nano-/microscale systems [25]. Chitosan is a natural biopolymer that is widely used in oral nanoparticulate formulations to provide increased drug concentration in the colon and to achieve improved therapy for the colon [26–28]. The degradation of chitosan occurs through the lysis of glycosidic bonds by the colonic microflora. It has been reported that nanoparticles prepared with polymers such as chitosan, whose surface charge is positive, remain longer in the mucus due to electrostatic interaction with the negative charge of the aqueous mucin layer [15,29–31].

Docetaxel (DCX) is obtained semi-synthetically from 10-deacetyl-baccatin isolated from the Taxus family (*T. baccata* and *T. brevifolia*). It is a potent and long-known anticancer agent that acts in the metaphase-anaphase process of cancer cells, exerts a cytotoxic effect on microtubules that are vital for mitotic cellular activity, and prevents the proliferation of cancer cells [32–34]. Its potent activity against a wide spectrum of cancers such as colon cancer, gastric cancer, breast cancer, recurrent ovarian cancer, and non-small cell lung cancer has been elucidated by in vitro and in vivo studies [35]. Its poor water solubility appears to be the primary problem and requires the addition of a co-solvent and/or a surfactant (ethanol/polysorbate 80) to the formulations [32,36]. However, results such as acute hypersensitivity reactions and decreased clinical efficacy have been reported due to auxiliary components such as Cremophor EL and polysorbate 80 included in the formulations. This situation requires routine premedication with antihistamines and/or glucocorticoids for patients to whom docetaxel will be administered [37–38]. While physicochemical problems such as insolubility can be overcome with innovative and rational formulation approaches, positive results in clinical efficacy and safety can be achieved by designs making use of the opportunities offered by new drug delivery systems [32,39–40].

In this study, orally administered PLGA nanoparticles were designed to be used against bowel tumors, and docetaxel was loaded into the nanoparticles as a model anticancer agent. CS coating was used to impart positive surface charge to negatively charged PLGA nanoparticles and to increase their interaction in the intestinal lumen. To date, an orally applicable and effective treatment approach to colon tumors has not been realized. Therefore, the main purpose of this study is to present an innovative approach to this problem. In this context, it is suggested that CS-coated PLGA nanoparticles with positive surface charge can provide stable and effective drug transport through the upper segments of the GIT to the colon after oral administration. The nanoparticles were designed to penetrate the colon tissue by showing higher local concentration in the tumoral region and to release the anticancer agent locally to a large extent. The aim of this study is to develop a new and unique orally administered approach for the treatment of colon tumors, to provide higher drug concentration in the tumoral region in the colon, to reduce systemic side effects compared to parenteral administrations, and to alleviate colon cancer. In vitro characterization, permeability studies, in vitro cell culture studies, and comprehensive release kinetics studies of the nanoparticular drug delivery system were carried out. It can be a detailed source and inspiration for possible future research in this area.

## Results and Discussion

### In vitro characterization of DCX-PLGA NPs and CS/DCX-PLGA NPs

Mean particle size, polydispersity index (PDI), and zeta potential of blank and DCX-loaded NPs are presented in Table 1. The mean particle size of the PLGA NPs was found to be in the range of 247.5–309.6 nm and the PDI ranged from 0.241 to 0.362, which is in the acceptable range (PDI < 0.4) for nanoparticular drug delivery systems [41]. The zeta potentials of blank and drug-loaded PLGA NPs were found to be −22.4 and −26.1 mV, respectively, while those of CS-coated PLGA NPs were +29.6 and +24.4 mV, respectively.

**Table 1:** Mean particle size, PDI, and zeta potential of blank and DCX-loaded formulations (*n* = 3, ±SD).

Nanoparticle formulations		Particle diameter ± SD (nm)	PDI ± SD	ZP ± SD (mV)

PLGA NPs	blank	247.5 ± 15.2	0.241 ± 0.036	−22.4 ± 2.2
	DCX-loaded	266.3 ± 14.2	0.322 ± 0.021	−26.1 ± 3.1
DCX-loaded	CS/PLGA NPs	287.8 ± 12.7	0.325 ± 0.029	+29.6 ± 3.2
	blank	309.6 ± 18.4	0.362 ± 0.041	+24.4 ± 2.2

As shown in Table 1, the CS coating on the NPs increased the particle size. The main reason of the increase in particle size is the electrostatic adsorption of CS macromolecules on the surface of the NPs [42]. Besides coating, drug loading to NPs also led to remarkable changes in particle size. Moreover, after CS coating, the zeta potential of PLGA NPs changed from negative to positive. The negative zeta potential is attributed to the presence of carboxyl groups of PLGA at the NP surface [43]. CS is a cationic heteropolysaccharide, which promotes cellular uptake, mucoadhesiveness, and tissue penetration [42]. Therefore, CS interacts easily with negatively charged groups at the surface of the NPs and increases the tissue penetration.

### Morphology of DCX-loaded nanoparticles

**Figure 1:**
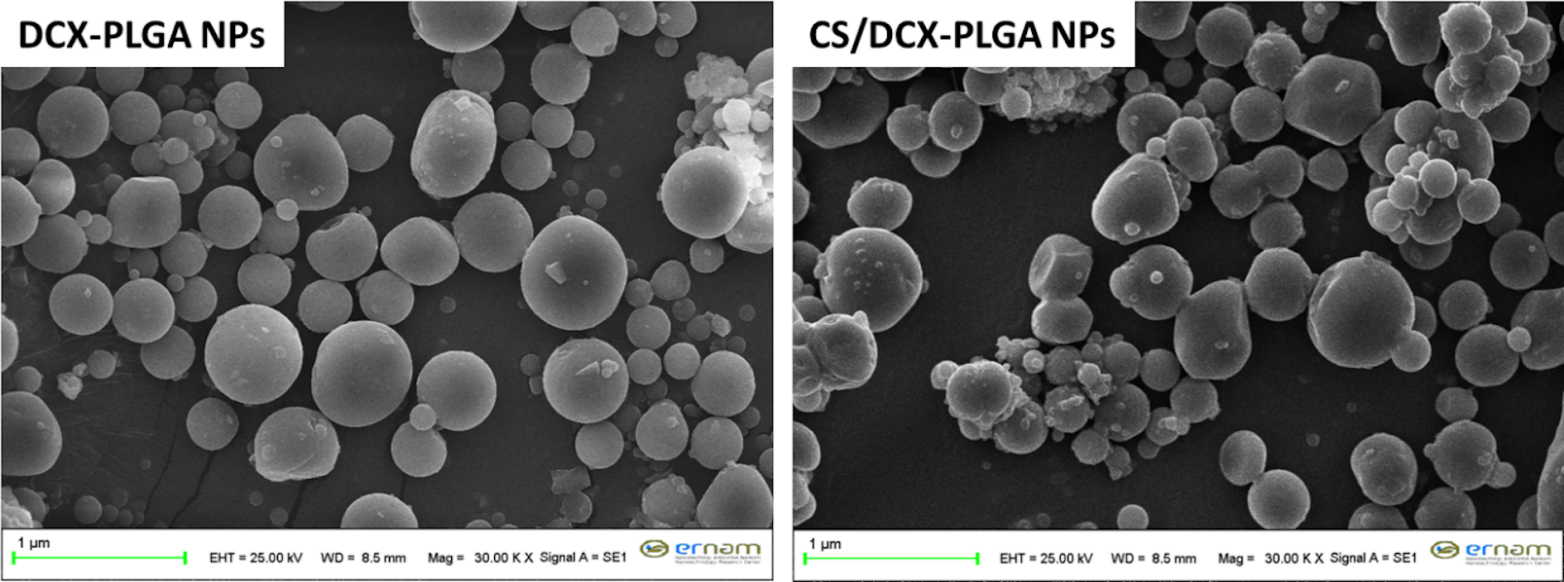
SEM images of the DCX loaded nanoparticle formulations.

Morphological properties of NPs are among the most important factors influencing the efficacy of drugs and determining the fate of NP systems. It has been reported that the NP morphology significantly affects circulation time and cellular uptake of NPs [44]. Morphological characterization of both coated and uncoated DCX-PLGA NPs was carried out by scanning electron microscopy (SEM). As it can be seen in Figure 1, both formulations exhibit perfectly round spheres with smooth surfaces. No free DCX crystals were found in the SEM pictures of any formulation, confirming that DCX was efficiently encapsulated in the NPs. In addition, SEM micrographs were interpreted to be in accordance with the mean particle size data measured with the dynamic light scattering.

### Determination of drug loading capacity

The rate of drug encapsulation is one of the important characterization parameters that affect the efficiency of NPs in the treatment of diseases. Therefore, a reliable and optimized encapsulation is crucial for drug delivery systems. Determining the encapsulation efficiency (EE) and drug loading (DL) is also the basic method to measure the number of drug molecules entrapped in the nanoparticles for experimental studies and dose calculations [45].

EE and DL of DCX-loaded nanoparticle formulations are documented in Table 2. The EE values of DCX-PLGA and CS/DCX-PLGA were 46.18% and 69.04%, respectively (*p* < 0.05). CS as a coating material led to an increase in encapsulation efficiency of the NPs. In the literature, there are several studies claiming that CS enhances the entrapment efficiency of NP systems [46–48]. While CS coats the surface of the NP, it also allows for the adsorption of the drug molecules on the NP surface [49]. Thus, NPs can entrap more drug molecules. Our results proved the positive effect of CS on EE and DL by showing consistency with results of previous studies.

**Table 2:** Encapsulation efficiency (EE), drug loading (DL) and production yield (PY) of DCX-loaded PLGA NPs (*n* = 3, mean ± SD).

Nanoparticle formulations	Encapsulation efficiency ± SD	Drug loading ± SD	Production yield % ± SD (PY)

DCX-PLGA NPs	46.2% ± 3.1%	5.1% ± 0.6%	81.8% ± 2.4%
CS/DCX-PLGA NPs	69.0% ± 4.9%	7.6% ± 0.8%	83.5% ± 4.6%

### In vitro release studies of DCX from nanoparticles

In order to mimic the GIT with regard to both pH and retention time, in vitro release studies for each formulation were carried out in three different media with different pH conditions (simulated gastric fluid (SGF): for 0–2 h in pH 1.2, simulated intestinal fluid (SIF): for 2–5 h in pH 6.8, and simulated colonic fluid (SCoF): for 5–72 h in pH 7.4). Thus, the release behavior of NPs was evaluated by simulating the GIT as close to reality as possible [10,26].

**Figure 2:**
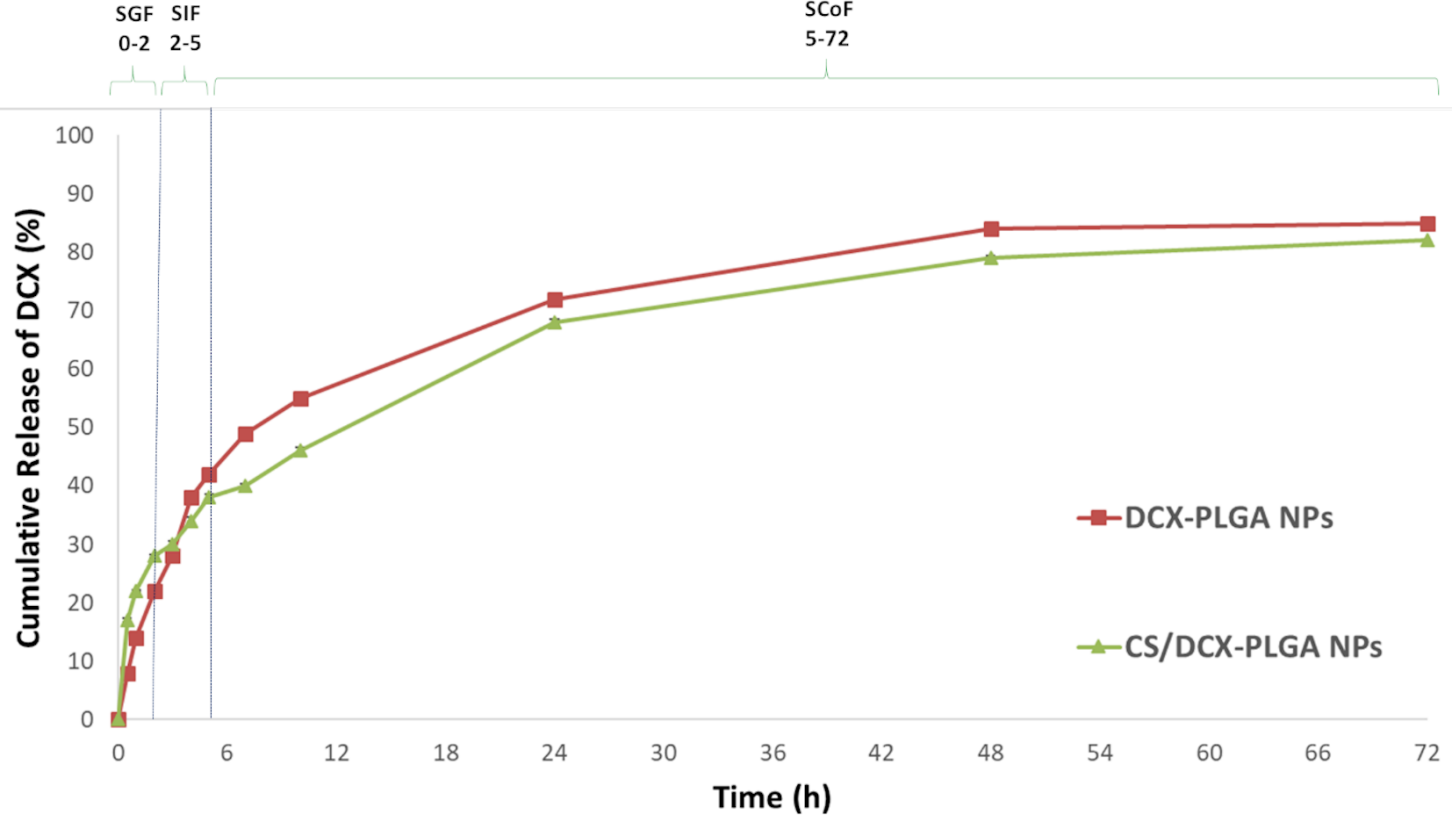
Cumulative in vitro release profile of DCX from formulations in simulated GIT fluids (*n* = 3, mean ± SD).

Figure 2 illustrates the release profile of both DCX-PLGA and CS/DCX-PLGA. In the first two hours, CS-coated NPs exhibited maximum DCX release and approximately 30% of the total entrapped drug was released from the NPs. Considering the first two hours, the main reason for the burst release profile is that CS on the NP surface absorbed the DCX molecules. The first interaction between the formulations and the low pH medium caused a release of DCX molecules attached to the CS surface of the NPs. Another reason why CS-coated PLGA NPs exhibited fast dissolution in acidic medium might be because CS degrades more easily in a low-pH environment [50]. After the first four hours, uncoated PLGA NPs began to release more DCX compared to the coated formulation. Until the end of the experiment, the release profile of CS-coated PLGA NPs remained slower than that of uncoated PLGA NPs. This situation was associated with the fact that the film layer formed on the nanoparticle surface with the CS coating causes a slower release after the burst effect. When both formulations reached the SCoF, they still retained an amount of more than 50% of the drug. The CS-coated PLGA formulation was able to preserve a DCX amount of about 60% and a substantial amount of DCX was released at the colonic pH values. It is known that surface modification with CS provides a prolonged drug release profile to NP [51–52].

### In vitro evaluation of nanoparticle interaction with mucus

Orally administered drug molecules have to cross several barriers in the GIT in order to exhibit an effect. Among them are mucosae, producing layers of complex aqueous mixtures covering epithelial surfaces including that of the GIT. For oral drugs, rapid elimination from the GIT by intestinal motility is among the most important obstacles for successful treatment [15]. The low permeability of nanoformulations through the mucus layer prevents sufficient absorption of the drug and causes clearance of molecules which do not reach adequate retention time in the GIT [53]. Thus, increasing the retention time of the NPs on the mucus layer enhances the therapeutic effect. The surface charge of NPs can be altered to enhance the interaction of NPs with the mucus layer. Luo et al. emphasized that interaction with the mucin layer, which is negatively charged owing to the sulfhydryl groups, could be enhanced by using positively charged particles [15]. Manca et al. stated that using CS as coating material significantly increased the mucoadhesive activity of the formulations by positively changing the surface charge of the NPs [54].

Cell culture models are not precise enough to evaluate the interaction between NPs and mucus layer. Since the interaction between NPs and mucus layer, which is the uppermost layer of the intestinal tract, is an important parameter, the mucoadhesive properties of formulations were evaluated. Artificial mucus layer and turbidimetric evaluation were employed to examine the nanoparticle interaction with mucus.

### Turbidimetric evaluation of mucin/nanoparticle interaction

**Figure 3:**
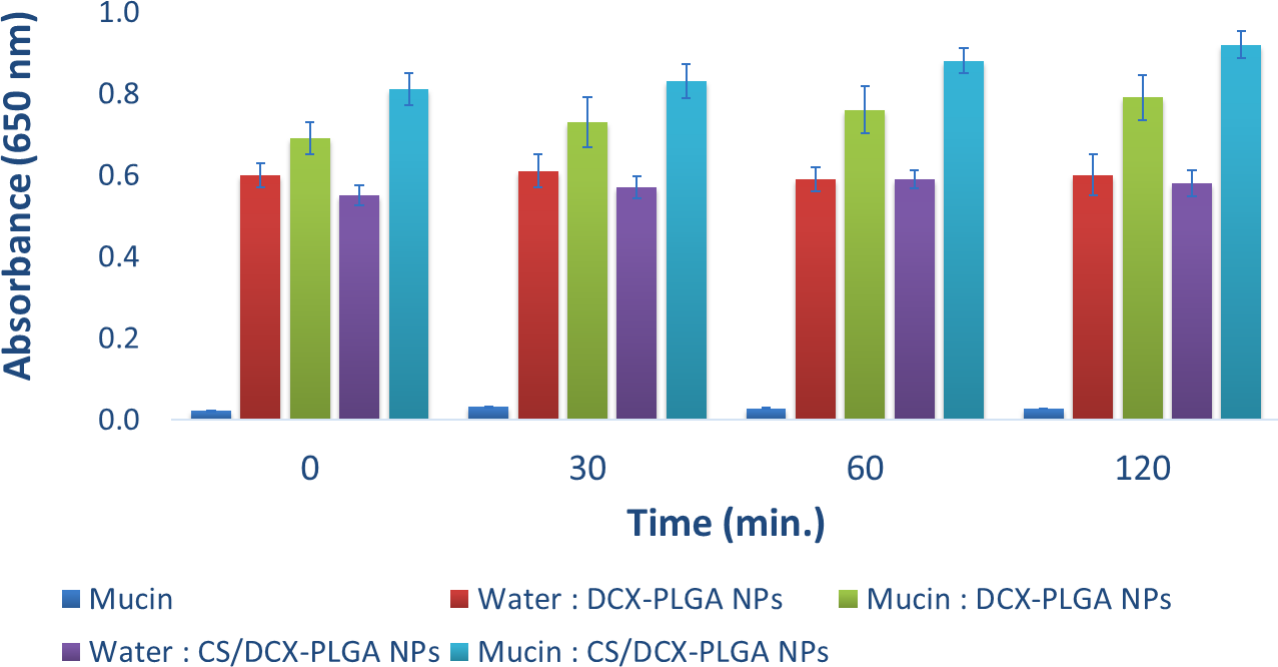
Turbidimetric evaluation of mucin/nanoparticle interaction at 650 nm (*n* = 3, mean ± SD).

Turbidimetric evaluation of mucin interaction was performed as described in the Methods section. Figure 3 shows data regarding the turbidimetric analyses. All nanoparticle formulations showed a tendency to interact with mucin. It is clearly seen that the absorbance of the CS-coated formulations was found to significantly increase compared to other formulations, which proves strong interaction between cationic CS and negatively charged mucin. This difference was statistically significant in CS-coated formulations (*p* < 0.05).

### Penetration of DCX-PLGA NPs and CS/DCX-PLGA NPs through an artificial mucus layer

**Figure 4:**
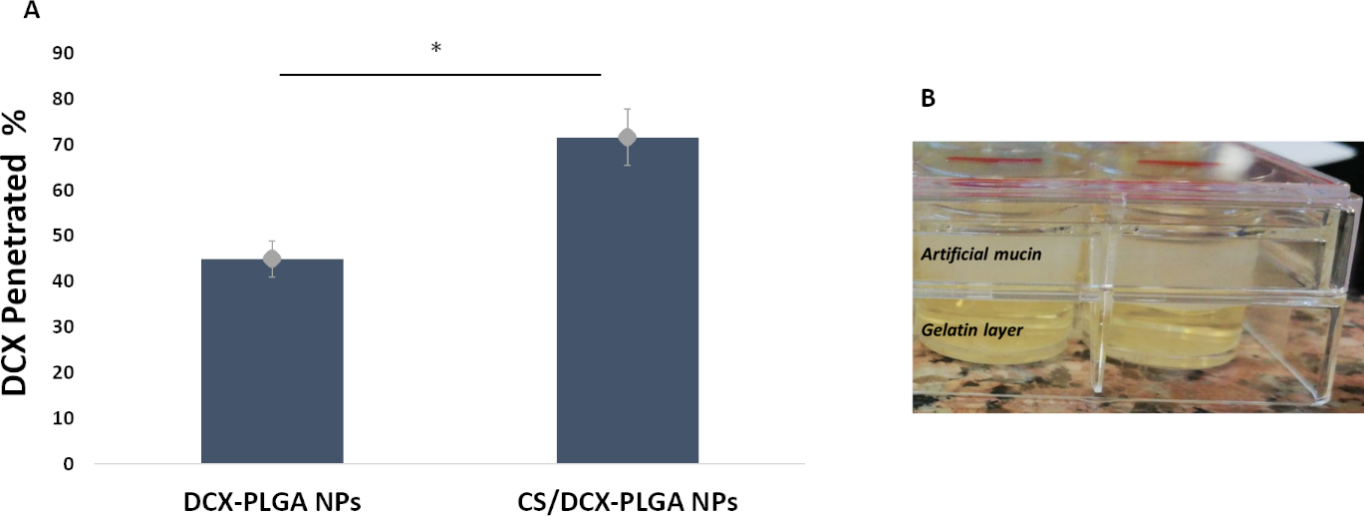
(A) Amount (%) of DCX penetrated through an artificial mucus layer. (B) Representative image of the experiment (*n* = 3, ± SD) (*; *p* < 0.05).

In order to evaluate the penetration capability of NPs, wells containing artificial mucus layer were treated with DCX-loaded NP formulations. Subsequently, NPs that had penetrated the mucus layer and moved into gelatin were measured using UV–vis spectrophotometry. The amount of penetrated DCX is summarized in Figure 4. The CS/DCX-PLGA formulation yielded the highest percentage of DCX penetration. Uncoated PLGA NPs penetrated the artificial mucus layer by 45%. Our results stated that surface charge has a significant effect on penetration through the mucus layer. Since the zeta potential of PLGA NPs changed from negative to positive due to CS coating, strong interaction occurred between NPs and mucus layer. In contrast, negatively charged unmodified PLGA NPs showed less interaction with the mucus layer since both mucus layer and PLGA surface have similar charge, resulting in repulsive forces between NPs and mucus layer [55].

### Release kinetics studies

**Figure 5:**
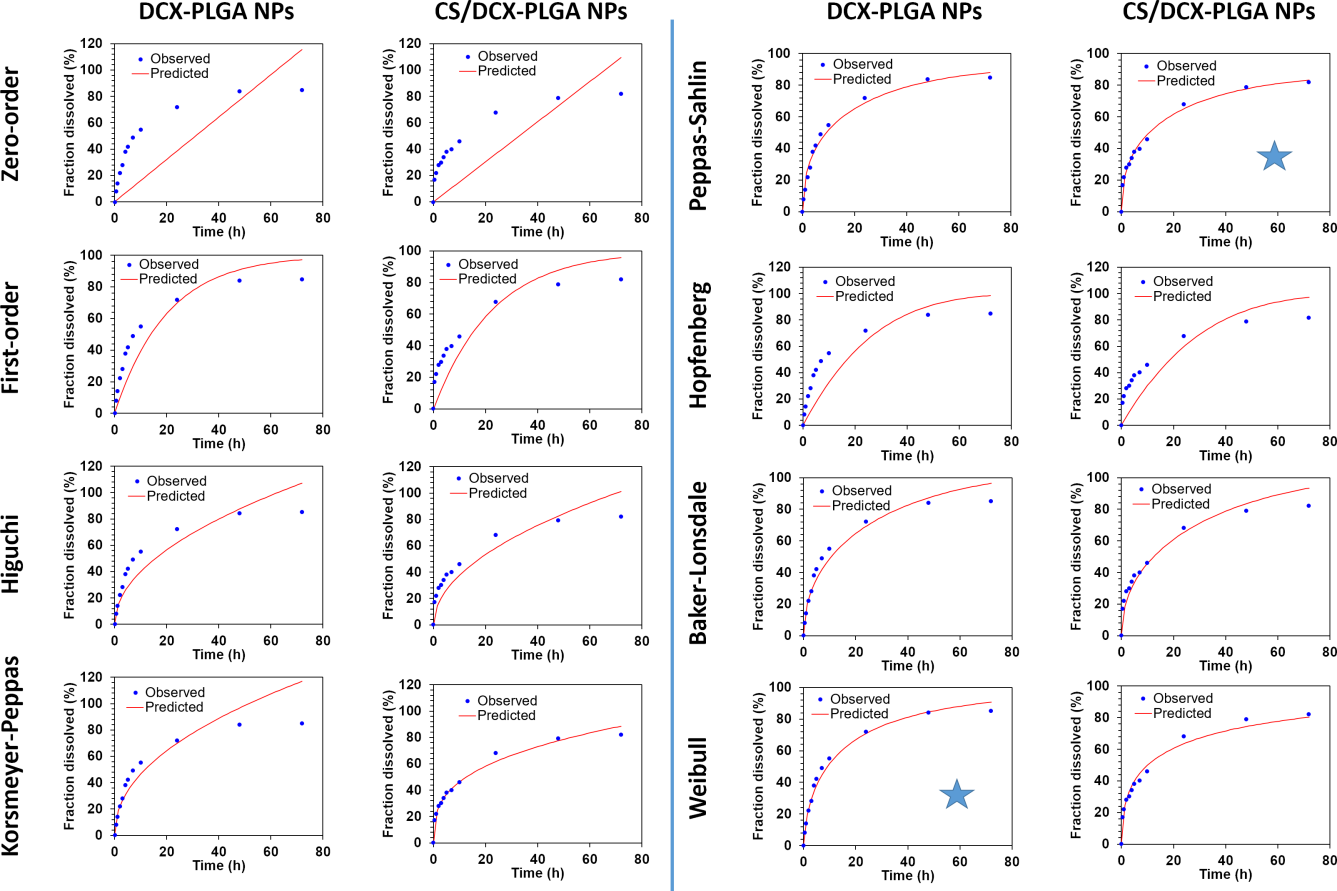
Release kinetics curves obtained with the DDSolver software for NPs (The blue stars indicate the best fit models).

There are several factors influencing the fate of therapeutical formulations. Release kinetics models are directly relevant for the efficacy and safety of the drugs [56]. Data obtained from in vitro release studies were quantitatively analyzed to determine kinetic models. The DDSolver software was used to determine four criteria (*R*^2^, *R*^2^_adjusted_, Akaike information criterion (AIC), and model selection criterion (MSC)), which help to investigate the mathematical models (zeroth order, first order, Higuchi, Korsmeyer–Peppas, Peppas–Sahlin, Hopfenberg, Baker–Lonsdale, or Weibull model). Many studies in this area only evaluate the in vitro release profile, but examining possible models in release kinetics, especially in oral drug delivery systems, is valuable for a clearer interpretation of release behavior. These quantitative evaluations help to accelerate the drug development processes by estimating the in vivo performance of formulations. The results of the release kinetics modelling studies are presented in Table 3 and Figure 5. In Figure 5, DCX release curves and the curves of the kinetics models are shown. There are overlaps between the release of DCX from DCX-PLGA NPs with the Weibull model and of the release of DCX from CS/DCX-PLGA NPs and the Peppas–Sahlin model. Furthermore, as seen in Table 4, the release profiles of DCX from the two different nanoparticle formulations were compared in terms of similarity (f2) and difference (f1) factors. The results reveal that the release profiles of nanoparticles, which we obtained using the same nanoparticle material, showed similar profiles [57–58].

**Table 3:** Release kinetic modeling and results of DCX-loaded PLGA nanoparticles.

Model and equation	Formulation	Evaluation criteria						

		Parameter		*R* ^2^	*R* ^2^_adjusted_	AIC	MSC	*n*/m*

zero-order	DCX-PLGA NPs	*k*_0_	1.606	0.0828	0.0828	110.2797	−0.2906	–
*F* = *k*_0_ · *t*	CS/DCX-PLGA NPs	*k*_0_	1.520	−0.0599	−0.0599	108.6661	−0.4964	–

first-order	DCX-PLGA NPs	*k*_1_	0.050	0.7673	0.7673	93.8232	1.0808	–
*F* = 100 · [1 − exp(−*k*_1_ · *t*)]	CS/DCX-PLGA NPs	*k*_1_	0.044	0.6408	0.6408	95.6818	0.5856	–

Higuchi	DCX-PLGA NPs	*k*_H_	12.627	0.8358	0.8358	89.6375	1.4296	–
*F* = *k*_H_ · *t*^0.5^	CS/DCX-PLGA NPs	*k*_H_	11.933	0.8201	0.8201	87.3856	1.2769	–

Korsmeyer–Peppas	DCX-PLGA NPs	*k*_KP_	15.806	0.8341	0.8176	91.7569	1.2530	0.468
*F* = *k*_KP_ · *t*^n^	CS/DCX-PLGA NPs	*k*_KP_	21.757	0.9870	0.9857	57.8898	3.7349	0.328

Peppas–Sahlin	DCX-PLGA NPs	*k*_1_	22.168	0.9768	0.9716	70.1584	3.0529	0.450
*F* = *k*_1_ · *t*^m^ + *k*_2_ · *t*^(2*m)^	CS/DCX-PLGA NPs	*k*_1_	20.994	0.9937	0.9923	51.1922	4.2931	0.450

Hopfenberg	DCX-PLGA NPs	*k*_HB_	0.008	0.6439	0.6083	100.9256	0.4889	4.500
*F* = 100 · [1 − (1 – *k*_HB_ * *t*)^n^]	CS/DCX-PLGA NPs	*k*_HB_	0.008	0.5174	0.4692	101.2245	0.1237	4.500

Baker–Lonsdale	DCX-PLGA NPs	*k*_BL_	0.005	0.9626	0.9626	71.8895	2.9086	–
3/2 · [1 − (1 − *F*/100)^(2/3)^] − *F*/100 = *k*_BL_ · *t*	CS/DCX-PLGA NPs	*k*_BL_	0.004	0.9544	0.9544	70.9086	2.6500	–

Weibull	DCX-PLGA NPs	β	0.594	0.9883	0.9857	61.9097	3.7403	–
*F* = 100 · {1 − Exp[−((*t* − Ti)^β^)/α]}	CS/DCX-PLGA NPs	β	0.430	0.9841	0.9806	62.2602	3.3707	–

**Table 4:** Calculation of the differences and similarities of the release profiles of the nanoparticles formulations with the difference (f1) and similarity (f2) factors.

Formulation	CS/DCX-PLGA NPs	
	
	difference factor (f1)	similarity factor (f2)

DCX-PLGA NPs	13.02	60.87

According to the release kinetics parameters, as seen in Table 3, the highest *R*^2^, *R*^2^_adjusted_, and MSC values, as well as the lowest AIC values were observed for the Weibull model for DCX-PLGA NPs, and for the Peppas–Sahlin model for CS/DCX-PLGA NPs. In the Weibull model, the exponent β (i.e., the shape parameter) is a parameter used to elucidate the release from a polymeric matrix. A value of β ≤ 0.75 indicates Fickian diffusion, while 0.75 < β < 1 indicates a combination of Fickian diffusion and controlled release [59]. The β value for the Weibull model was calculated as 0.594 for the DCX-PLGA NPs. According to the literature, when these data are examined within the scope of the Weibull model, the DCX release kinetics from DCX-PLGA nanoparticles were found to be compatible with Fickian diffusion [60]. This shows that in the model-dependent baseline evaluation of in vitro release profiles, the drug adsorbed on the nanoparticle surface or encapsulated in the nanoparticle material is released from the polymeric structure on the basis of diffusion as the major mechanism. It has been confirmed by mathematical modeling that the release is based on diffusion [10,61]. In contrast, the Peppas–Sahlin model describes the drug release from CS/DCX-PLGA NPs. The Peppas–Sahlin model is based on the combination of diffusion and erosion of the nanoparticle matrix. In order to further elucidate the kinetics model, a few more parameters were examined [62]. The diffusional exponent values (*n* or *m*) regarding the release kinetics from the nanoparticles were computed. The diffusional exponent indicating the drug release mechanism is represented by “*n*” in the Korsmeyer–Peppas model while “*m*” represents the same parameter in the Peppas–Sahlin model [63]. In order to examine the Peppas–Sahlin model more deeply, the diffusional exponent value *m* was computed as 0.450 for the CS/DCX-PLGA NPs. A value of *m* < 0.45 indicates Fickian diffusion. A value of 0.45 < *m* < 0.85 shows that the drug release occurs through non-Fickian diffusion. A quotient of *m*/*n* = 0.85 is consistent with a case-II transport and *m*/*n* > 0.85 indicates super-case-II transport [63–66]. Here, the diffusional exponent parameters of the Peppas–Sahlin model are consistent with Fickian diffusion. Thus, it was determined that basically diffusion-based drug release occurred in both formulations. However, results indicated that the release from DCX-PLGA and CS/DCX-PLGA formulations is described by different models although both formulations have similar dissolution profiles. The main difference between the two formulations is the CS coating. The release profiles were different at the beginning, and a faster release was observed from CS-coated nanoparticles. However, in the following process, the film layer of CS on the nanoparticle surface led to a slower release. All results together indicate a release based on Fickian diffusion.

### In vitro cell culture studies

#### Antiproliferative effect of DCX loaded PLGA nanoparticles against HT-29 cell line

**Figure 6:**
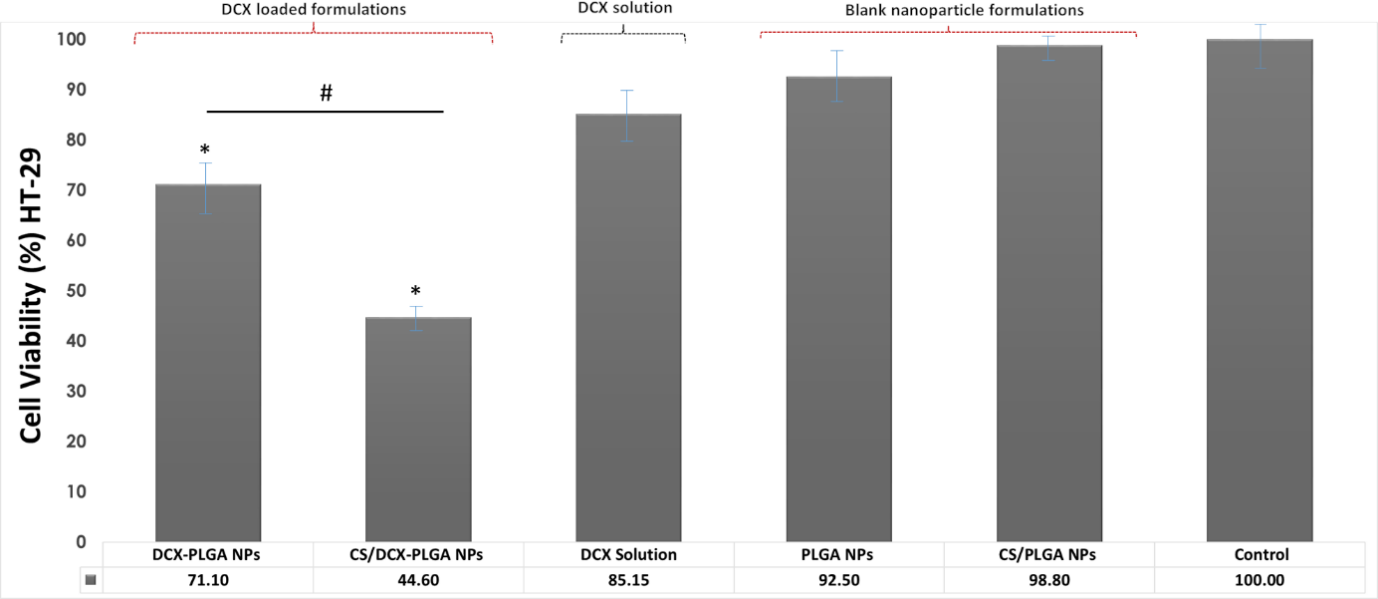
Anticancer activity of DCX-loaded and blank PLGA nanoparticles and free DCX on HT-29 cell line after 48 h treatment (*n* = 6, mean ± SD) (# *p* < 0.05 and * *p* < 0.05 compared with DCX solution).

The antiproliferative activity of DCX-loaded and blank NPs was investigated on the HT-29 human colon cancer cell line. DCX-loaded and blank NPs were compared to equivalent concentrations of DCX solution for 48 h incubation time and data are shown in Figure 6. For the analysis of the antiproliferative activity of the NP formulations, an incubation time of 48 h was chosen considering the doubling time and the in vitro release profiles of the NPs [10].

PLGA NP formulations loaded with DCX have been found to show considerable toxicity on HT-29 colon cancer cells. As illustrated in Figure 6, the HT-29 cell line treated with DCX-PLGA NPs and CS/DCX-PLGA NP formulations show 71.1% and 44.6% cell viability, respectively, whereas the cell viability of the control with DCX solution was more than 85%. DCX-loaded NP formulations showed significantly higher anticancer activity compared to DCX solution and blank NP formulations after the same incubation time and at the same concentration (*p* < 0.05). Additionally, CS/PLGA NPs exhibited a higher anticancer activity than DCX-PLGA formulations (*p* < 0.05). It is suggested that this is related to the positively charged surface of the CS-coated PLGA NPs and a strong cellular interaction resulting in increased cellular uptake.

In the literature, there are various studies showing consistency with our results. Varan et al. reported that cationic nanoparticles have a higher tendency to interact with the negatively charged cell membrane [67]. Accordingly, Verma et al. stated that the surface properties and charges of nanoparticles play an essential role in the interaction between nanoparticles and cell membrane and the subsequent intracellular fate of the nanoparticles [68]. Similarly, Chen et al. revealed that PLGA NPs coated with CS had higher anticancer activity then unmodified formulations [69].

When DCX-loaded PLGA NPs were compared to DCX solution, the NP formulations exhibited considerably higher antiproliferative activity. This was due to the increased uptake of the nanoscale particles by the cells. In general, similar results and high anticancer activity are seen in nanoscale drug delivery systems. The higher cytotoxicity is related to an increase in the amount of drug that the nanoparticles can carry into the cell. CS modification increased the anticancer activity of the NP formulation by a factor of about 1.5.

#### In vitro intestinal permeability of NPs across Caco-2 cell line

Intestinal permeability studies of DCX-loaded NPs and free DCX were carried out using the Caco-2 cell line. Drug molecules have to overcome several barriers throughout the GIT, including the mucus layer, intestinal epithelial cells, and the endothelium of the capillaries. Among them, the monolayer of epithelial cells plays an essential role in the absorption of drugs. Therefore, the Caco-2 cell line derived from a human colorectal carcinoma was employed to simulate the epithelial cell layer. A monolayer of cells should have a transepithelial electrical resistance of around 500 Ω·cm^2^ to show similarity with intestinal lumen [70–72].

In Table 5, the apparent permeability coefficient (*P*_app_) values of free DCX and DCX-loaded NP formulations are presented. The apparent permeability coefficient (*P*_app_) value for free DCX was 0.91 ± 0.049 × 10^−6^ cm/s. The *P*_app_ values of DCX-PLGA NPs and CS/DCX-PLGA NPs were 1.22 ± 0.136 × 10^−6^ cm/s and 3.49 ± 0.421 × 10^−6^ cm/s, respectively. Both nanoparticulate DCX formulations exhibited a significantly enhanced permeation across the cell line compared to free DCX (*p* < 0.05). Moreover, DCX-PLGA NPs increased *P*_app_ by 134.1% while CS/DCX-PLGA NPs increased *P*_app_ by 383.5%. The *P*_app_ of CS/DCX-PLGA NPs was significantly higher than that of DCX-PLGA NPs (*p* < 0.05). The reason why the CS-coated formulation has the highest *P*_app_ is that CS modification leads to a positively charged surface of the NPs with increased retention time and cellular uptake. In order to assess the effect of surface charge on penetration, Ünal et al. examined the transportation of the positively and negatively charged NPs loaded with DCX through the Caco-2 cell layer. Cationic NPs showed an approximately 50% increase in penetration in comparison with anionic NPs [73]. In another study, Sheng et al. investigated the permeation of CS-coated PLGA NPs. As a result, CS-coated PLGA NPs improved the oral absorption and remarkably increased the cellular uptake compared to unmodified PLGA NPs [74].

**Table 5:** Apparent permeability coefficient (*P*_aap_) of DCX on Caco-2 cell monolayer (*n* = 3, ±SD).

Formulation	*P*_app_ (× 10^−6^ cm/s) ± SD	Increase in permeability (%)

free DCX	0.91 ± 0.05	–
DCX-PLGA NPs	1.22 ± 0.14	134.1
CS/DCX-PLGA NPs	3.49 ± 0.42	383.5

## Conclusion

Currently, many anticancer agents used in chemotherapy are administered parenterally. Significant advances in the field of oral chemotherapy might lead to a new era in chemotherapy. Long-term chemotherapy in chronic and high-mortality cancers, such as bowel cancers, bring with them a series of problems in terms of patient compliance and burden on the health system. Also, bowel cancers occur in the farthest part of the GIT, presenting a greater challenge for oral formulations than many other types of cancer. Because of the transit time, variable pH, and enzymatic activity of the GIT, as well as the presence of mucin in the bowel region resulting in the clearance of the drug, rational formulation designs need to be developed. In this study, it was aimed to deliver DCX to the bowel effectively and stably through oral administration of CS-coated PLGA NPs. The CS/DCX-PLGA oral formulation, whose extensive in vitro characterization studies have been completed, showed high mucin interaction, high mucus penetration, high anticancer activity in a colon cancer cell line, and high intestinal permeability. Upon completion of extensive mathematical release kinetic analyses, the proposed formulation can be recommended as an oral formulation for bowel cancer. It is considered that the results of this study will shed light on future studies.

## Experimental

### Materials

Poly(lactide-*co*-glycolide) (PLGA) 50:50 (RG 502H) (*M*_w_ = 7–17 kDa; lactide/glycolide = 50:50) was purchased from Sigma-Aldrich (St. Louis, MO, USA). Chitosan (Protasan UP G-113; *M*_w_ < 200 kDa) was purchased from Novamatrix, Norway. Docetaxel was kindly donated by ILKO, Turkey. Ethyl acetate, dialysis cellulose tubing membrane (average flat width 25 mm, MWCO 14,000 Da), gelatin type B from bovine skin, mucine from porcine stomach (type II), diethylenetriaminepentaacetic acid (DTPA; min 99%, titration), and egg yolk emulsion were purchased from Sigma-Aldrich, USA. All other chemicals used were of analytical grade and obtained from Sigma-Aldrich.

### Methods

#### Preparation of DCX-PLGA and CS/DCX-PLGA NPs

DCX-PLGA NPs and CS/DCX-PLGA NPs were prepared by the previously reported single-emulsion preparation method, with some modifications [75–76]. In accordance with the principle of the method, the organic and aqueous phase were prepared separately. First, the organic phase was prepared by dissolving 2% (w/v) PLGA and 20 mg docetaxel in 10 mL ethyl acetate. 25 mL of aqueous phase was prepared by dissolving the non-ionic surfactant PVA at a concentration of 2% and CS at a concentration of 0.2% [77]. The organic phase was added to the aqueous phase on a magnetic stirrer at 550 rpm. The resulting o/w emulsion was sonicated on an ice bath with an ultrasonic probe at 25% power for 1 min (four times at 10 s intervals), and PLGA nanoparticles were obtained. The nanoparticles were stirred continuously for 24 h with a magnetic stirrer and the organic phase was evaporated. Then the PLGA nanoparticles were precipitated by centrifugation at 10000 rpm for 45 min and washed four times with distilled water. DCX-PLGA NPs pellets were suspended in 2 mL of solution containing 5% (w/w) mannitol, frozen at −80 °C, lyophilized (Labconco, USA) and stored at +4 °C until the experimental procedures. Similarly, CS/DCX-PLGA NPs were lyophilized by adding mannitol (5% w/v) as a cryoprotectant for further characterization. All procedures were followed similarly, except for the addition of DCX to the organic phase for the blank PLGA nanoparticles.

### In vitro characterization of the nanoparticles

#### Mean particle size and surface charge

Mean particle size (nm), PDI, and zeta potential (mV) of the NPs were investigated by dynamic light scattering (DLS; Malvern Zetasizer Nano ZS series, UK) with a disposable capillary cell. Particle size measurements were made at an angle of 173°, while zeta potential measurements were made at an angle of 12.8°. All formulations were measured at 25°C in triplicate. The particle size distribution was expressed as mean diameter (nm) ± standard deviation (SD) and PDI. The zeta potential (mV) was stated as the average of three subsequent measurements ± SD.

#### Particle shape and surface morphology

The shape and surface morphology of DCX-PLGA NPs and CS/DCX-PLGA NPs were investigated by SEM (Zeiss evo LS-10, Germany). For this purpose, NPs were covered with a 100 Å thick coating of gold and palladium and inserted on metal stubs before being dried for a 24 h SEM analysis.

#### Determination of encapsulation efficiency, drug loading and production yield

The previously reported UV–vis spectrophotometric quantification method was used to determine the DCX encapsulation efficiency of the prepared nanoparticles [78]. Encapsulated DCX was extracted from freeze-dried NPs formulations. Briefly, drug and polymer were dissolved by adding 1 mL of methanol to the lyophilized nanoparticles. This mixture was vortexed for 1 min, and then bath sonication was applied for 1 min. This mixture was then centrifuged at 6000 rpm for 15 min. The drug-containing supernatant was separated and stored. To prevent methodological drug loss, 1 mL of ethanol was added to the precipitate and the same procedure was repeated. Then, the first and second supernatants were mixed. The supernatant was analyzed and the amount of DCX was measured by UV spectrophotometry (Shimadzu UV-1800 UV–vis spectrophotometer, Shimadzu corporation, Japan) at 230 nm (λ_max_). Validation of the spectrophotometric method was carried out. Linearity, accuracy, precision, reproducibility, limit of detection (LOD), and limit of determination (LOQ) were determined for validation of the spectrophotometric method used for DCX quantification. Absorbance values for the amount of DCX were converted to concentration according to the standard calibration curve (*r*^2^ = 0.994). LOD and LOQ values were calculated as 0.0173 mg/mL and 0.0634 mg/mL, respectively. All experiments were repeated three times, and mean values were used. Encapsulation efficiency (EE), drug loading (DL), and production yield (PY) of nanoparticles were calculated using the following equations:


[1] $$\begin{array}{l} \text{encapsulation efficiency}\left(\text{EE}\right)\%=\\ \frac{\text{entrapped DCX amount in NPs}}{\text{theoretical DCX added to organic phase}}\times 100 \end{array}$$

[2] $$\begin{array}{l} \text{drug loading}\left(\text{DL}\right)\%=\\ \frac{\text{entrapped DCX amount in NPs}}{\text{total weight of NPs}}\times 100 \end{array}$$

[3] $$\begin{array}{l} \text{production yield}\left(\text{PY}\right)\%=\\ \frac{\text{total weight of NPs}}{\text{total amount of PLGA and DCX}}\times 100 \end{array}$$

#### In vitro release study

The in vitro release profile of DCX from DCX-PLGA NPs and CS/DCX-PLGA NPs was examined in a release medium with progressively changing pH, reflecting the GIT environment. The real transit periods were examined with the dialysis membrane technique at 37 °C in a shaking water bath (100 rpm) under sink circumstances [10]. Release experiments were conducted by placing 3 mL DCX-loaded NP formulation (weight equivalent to 5 mg DTX) in the dialysis membrane with a molecular weight cut off of 8–14 kDa (Sigma-Aldrich, USA). The dialysis membrane was immersed in the following release media representing the GIT pH values and transit times that the formulation would encounter after oral administration at 37 °C: simulated gastric fluid (SGF) pH 1.2 for a period of 0–2 h, followed by simulated intestinal fluid (SIF) for pH 6.8 for a period of 2–5 h, and finally simulated colonic fluid (SCoF) pH 7.4 for a period of 5–72 h. The dialysis membrane bag was transferred to the following release medium at the end of each appropriate time point. 1 mL of sample was taken from the dialysis bag at predetermined time points (0.5, 1, 2, 3, 4, 5, 7, 10, 24, 48, and 72 h) and replaced by an equal volume of fresh release medium, pre-heated to 37 °C, to conserve sink conditions. The cumulative percentage of total DCX released for each time point was quantified by using UV–vis spectrophotometry as described above.

### In vitro evaluation of nanoparticle interaction with mucus

#### Turbidimetric evaluation of mucin/particle interaction

In order to evaluate the mucoadhesive tendency of nanoparticles, the interaction of mucin and DCX-loaded nanoparticle formulations was evaluated spectrophotometrically by turbidity measurements at 650 nm [10,14,79]. The basis of the experiment is to compare the absorbance of the mucin solution at 650 nm as a reference and the absorbance of the mixture of nanoparticle aqueous dispersion and the mucin. For this purpose, 40 mg mucin powder was dispersed in 50 mL ultrapure water and stirred for 12 h. Then, by centrifugation at 8000 rpm for 15 min, excess mucin was removed and the mucin solution was obtained. Mucin solution and nanoparticle dispersions were mixed at a ratio of 1:4 (mucin solution/nanoparticle dispersion, v/v) and vortexed for 90 s. Mucin–nanoparticle mixtures and aqueous dispersions of nanoparticles were incubated at 37 °C and turbidimetric measurements were conducted at 650 nm at predetermined time points (0, 30, 60, and 120 min).

#### Penetration of DCX-PLGA NPs and CS/DCX-PLGA NPs through artificial mucus layer

The penetration ability of DCX-PLGA NPs and CS/DCX-PLGA NPs through an artificial mucus layer model (AMLM) was conducted as previously reported [79]. This technique involved the formation of an artificial mucus layer, which was then applied over the gelatin layer. Initially, a 10 percent (w/v) gelatin dispersion was made by heating 50 mL of ultrapure water on a magnetic stirrer to 60 °C, and then 1 mL of the dispersion was added to each well of the 24-well cell plates. The gelatin layer in the experimental model was cooled to room temperature. 250 mg of mucin, 0.295 mg of diethylenetriamine pentaacetic acid (DTPA), 250 mg of sodium chloride, 110 mg of potassium chloride, 250 μL of sterile egg yolk emulsion, and 1 mL of RPMI were dissolved in 50 mL of ultrapure water while being magnetically stirred at room temperature to prepare artificial mucus. In the 24-well plates, 1 mL of artificial mucus dispersion was applied to each well on the gelatin layer. The placement of the artificial mucus solution to the gelatin layer completes the construction of the artificial mucus layer model with which the experiments were conducted.

In order to evaluate the penetration of formulations, 500 μL of NP dispersion was added onto this artificial mucus model and left for incubation for 24 h at room temperature. The artificial mucus containing NPs was removed from each well after 24 h. To completely clean the mucus, the gelatin layer was washed with ultrapure water (2 mL × six replicates). The solid gelatin was then heated to 60 °C in order to liquefy again. The supernatant was then collected after centrifuging the sample for 25 min at 5500 rpm. The validated quantification method was used to determine the amount of DCX in the supernatant. Thus, the ability of NPs to penetrate first through the mucus layer and then reach the gelatin layer was assessed by measurement of the amount of DCX transported into the gelatin layer.

#### Release kinetics study

In vitro release profiles of DCX from DCX-PLGA NPs and CS/DCX-PLGA NPs formulations were assessed with the DDSolver software. In this context, several mathematical models were applied to analyze DCX release kinetics from NPs (zeroth order, first order, Higuchi, Korsmeyer–Peppas, Peppas–Sahlin, Hopfenberg, Weibull, and Baker–Lonsdale). In vitro release data were processed using the DDSolver program to identify four criteria for the selection of the “best fit” models, that is, coefficient of determination (*R*^2^), adjusted coefficient of determination (*R*^2^_adjusted_), Akaike information criterion (AIC), and model selection criterion (MSC). The models to fit the in vitro release data were identified using the highest *R*^2^, *R*^2^_adjusted_ and MSC values, and the lowest AIC values [80–81]. Additionally, using a model-independent method, the similarities and differences between DCX release profiles from DCX-PLGA NPs and CS/DCX-PLGA NPs were assessed in accordance with the “similarity (f2)” and “difference (f1)” factors [80,82]. The FDA’s “Center for Drug Evaluation and Research's Guidance for Industry” was used to calculate difference factor (f1) and similarity factor (f2) in order to compare the release profiles of formulations (CDER) [83]. Equation 4 and Equation 5 were used for the calculation of f1 and f2 factors [84]. Based on f1 values between 0 and 15 and f2 values between 50 and 100, it is noted that the two release profiles seem to be similar [85].


[4] $$f1=\left\{\left(\sum_{t=1}^{n}\left|R-T\right|\right)/\left(\sum_{t=1}^{n}R\right)\right\}\times 100$$

[5] $$f2=50\cdot \log\left[\frac{100}{\sqrt{1+\frac{\sum_{t=1}^{n}{\left[Rt-Tt\right]}^{2}}{n}}}\right]$$

### In vitro cytotoxicity studies

Using the colorimetric assay technique, the cytotoxicity of DCX-PLGA NPs and CS/DCX-PLGA NPs was assessed. A cell culture study approach was used in this case to compare the activity of drug-loaded nanoparticles with an equivalent amount (5 µg/mL) of DCX solution in DMSO for 48 h on the HT-29 human colon cancer cell line. HT-29 cells were seeded in 96-well plates (10^4^ cells/100 µL per well) and incubated for 24 h to attach the cells in the wells. Samples were diluted with medium (DMEM containing 10% BSA) equivalent to 5 μg/mL DCX in 100 µL for either DCX solution or nanoparticle formulations (blank and DCX-loaded). The cells were incubated with formulations and drug solution for 48 h.

After incubation, water-soluble tetrazolium salt (WST-1) (10 µL) was added to wells and incubated at 37 °C for 4 h. Then, the optical density (OD) was measured via a cell plate reader at 450 nm (BiotEKM Synergy HT, USA). Cell viability (%) was calculated according to the following equation:


[6] $$\text{cell viability }\left(\%\right)=\left(\frac{\text{OD of treated wells}}{\text{OD of untreated cells}}\right)\times 100$$

#### Evaluation of in vitro transport of DCX across the Caco-2 cell line

In vitro transport studies of DCX with DCX-PLGA NPs and CS/DCX-PLGA NPs were conducted across the Caco-2 cell line (human adenocarcinoma cells) (HTB-37™, ATCC, USA). Caco-2 cells, ninth passage, were used for this analyses. Before the experiment, cells were seeded onto the apical side of 12-well polycarbonate membrane filters at a density of 60,000 cells/insert (Thincerts™, pore diameter 1 μm, area 1.13 cm^2^). The inserts were loaded with 500 µL of medium for the apical side and 1000 µL of medium for the basolateral side of the wells. Media were replaced every 48 h for 21–25 days and cells were incubated in an incubator at 37 °C with 5% CO_2_ supply. Before performing the transmembrane permeability studies, the Caco-2 cell monolayer’s integrity was confirmed by measuring the transepithelial electrical resistance (TEER). Transmembrane permeability analyses were begun when TEER values reached around 500 Ω·cm^2^ on the 22th day (524 ± 31 Ω·cm^2^) of the cell seeding [86]. The TEER value was determined using an epithelial volt/ohm meter (EVOM^2^) (WPI Wolrd Precision Instruments, USA).

The apical and basolateral compartments were washed three times with Hank's balanced salt solution (HBSS) (preheated to 37 °C). DCX solution, DCX-PLGA NPs, and CS/DCX-PLGA NPs were diluted to an equivalent concentration of 40 µg/mL DCX. Subsequently, DCX solution and DCX-loaded nanoparticle formulations were placed to the apical side prepared in 0.5 mL HBSS, and 1 mL free HBSS was added to the basolateral region.

The cell monolayer was incubated with the formulations at 37 °C for 4 h. At predetermined time intervals, 0.3 mL samples were taken from the basolateral side and replaced with fresh medium. For each sample, the DCX amount was analyzed by validated UV–vis spectrophotometric quantification. All experiments were conducted in triplicate. The following equation was used to calculate the apparent permeability coefficients (*P*_app_, cm/s) for each experimental group:


[7] $$P_{\text{app}}=\frac{\text{d}Q}{\text{d}t}\cdot \frac{1}{A\cdot C_{0}}$$

where *A* is the monolayer’s surface area, *C*_0_ is the initial concentration on the apical side, and d*Q*/d*t* is the permeability rate.
